# Spatial pattern of genetic diversity and selection in the MHC class II *DRB* of three Neotropical bat species

**DOI:** 10.1186/s12862-016-0802-1

**Published:** 2016-10-26

**Authors:** Arielle Salmier, Benoit de Thoisy, Brigitte Crouau-Roy, Vincent Lacoste, Anne Lavergne

**Affiliations:** 1 Laboratoire des Interactions Virus-Hôtes, Institut Pasteur de la Guyane, 23 avenue Pasteur, BP 6010, 97306 Cayenne, Cedex French Guiana; 2 CNRS, Université Toulouse 3 UPS, ENFA, UMR 5174 EDB (Laboratoire Évolution et Diversité Biologique), 118 Route de Narbonne, 31062 Toulouse, France

**Keywords:** MHC, *DRB* polymorphism, Selection, *Carollia perspicillata*, *Desmodus rotundus*, *Molossus molossus*

## Abstract

**Background:**

Although bats are natural reservoirs of many pathogens, few studies have been conducted on the genetic variation and detection of selection in major histocompatibility complex (MHC) genes. These genes are critical for resistance and susceptibility to diseases, and host–pathogen interactions are major determinants of their extensive polymorphism. Here we examined spatial patterns of diversity of the expressed MHC class II *DRB* gene of three sympatric Neotropical bats, *Carollia perspicillata* and *Desmodus rotundus* (Phyllostomidae), and *Molossus molossus* (Molossidae), all of which use the same environments (e.g., forests, edge habitats, urban areas). Comparison with neutral marker (mtDNA D-loop) diversity was performed at the same time.

**Results:**

Twenty-three *DRB* alleles were identified in 19 *C. perspicillata*, 30 alleles in 35 *D. rotundus* and 20 alleles in 28 *M. molossus*. The occurrence of multiple *DRB* loci was found for the two Phyllostomidae species. The *DRB* polymorphism was high in all sampling sites and different signatures of positive selection were detected depending on the environment. The patterns of *DRB* diversity were similar to those of neutral markers for *C. perspicillata* and *M. molossus.* In contrast, these patterns were different for *D. rotundus* for which a geographical structure was highlighted. A heterozygote advantage was also identified for this species. No recombination or gene conversion event was found and phylogenetic relationships showed a trans-species mode of evolution in the Phyllostomids.

**Conclusions:**

This study of MHC diversity demonstrated the strength of the environment and contrasting pathogen pressures in shaping *DRB* diversity. Differences between positively selected sites identified in bat species highlighted the potential role of gut microbiota in shaping immune responses. Furthermore, multiple geographic origins and/or population admixtures observed in *C. perspicillata* and *M. molossus* populations acted as an additional force in shaping *DRB* diversity. In contrast, *DRB* diversity of *D. rotundus* was shaped by environment rather than demographic history.

**Electronic supplementary material:**

The online version of this article (doi:10.1186/s12862-016-0802-1) contains supplementary material, which is available to authorized users.

## Background

Regarding adaptation to infectious diseases, genes of the major histocompatibility complex (MHC) are the most commonly studied in mammals. MHC genes play a significant role in the functionality and effectiveness of the immune response of vertebrates and are directly involved in fitness and adaptation of hosts to their pathogens [1]. Therefore, studying MHC gene diversity in non-model animals, known to be reservoirs of pathogens, is a critical tool to assess its evolutionary process and implication in (1) the variation of individual fitness, (2) population viability and (3) evolutionary potential in a changing environment [2]. Studies have essentially focused on the genetic variability of exon 2 of the MHC class II *DR beta* (*DRB*) gene given that this exon encodes the peptide-binding region (PBR) [1]. Hence, most of the variability displayed is recorded on this exon. Variations within the PBR define the repertoire of antigenic determinants and subsequently the ability to recognize a variable number of circulating pathogens [3]. Optimum resistance to pathogens is, therefore, the result of all intrinsic and extrinsic factors that influence the genetic variability of individuals’ immune system [4]. Most of the vertebrate populations studied so far exhibited high levels of MHC diversity both in the number of alleles and in their heterozygosities and allelic variation [5–8]. Pathogen-driven balancing selection and sexual selection seem to play a fundamental role in the preservation of this high level of polymorphism [2]. Pathogen-driven balancing selection acts through antagonistic host–parasite coevolution *via* several mechanisms: overdominant and frequency-dependent selection [9], as well as spatial and temporal variation in host pathogens [10]. Sexual selection pressures encompass mechanisms such as maternal–fetal interactions [11] and mate selection [12]. However, when assessing the genetic variability of both neutral markers and MHC genes, studies have highlighted the role of past demographic processes (e.g., fragmentation, bottlenecks, geographic isolation) in shaping the pattern of MHC variability that can sometimes surpass that of natural selection [13–18].

Local immunogenetic adaptation of hosts that live in different environments was associated with different parasite and pathogen pressures [19–22]. Indeed, differences in the diversity of pathogens (inducing different selection pressures on the hosts) are directly linked to environmental components. These latter (e.g., vegetation cover and density, landscape fragmentation, human occupation) modulate parasite and pathogen species richness, their survival and adaptability, as well as their distribution, transmission, developmental success and their ability to induce diseases [23]. Environmental components likewise impact the richness, population dynamics, immunocompetence and nutritional status of host species, all of which subsequently determine resistance or susceptibility to disease [24, 25]. A strong correlation was also shown between host and parasite species richness, their life history and ecological traits [26–28]. Moreover, anthropogenic alterations of habitats induce changes in host–pathogen–environment interactions and are consequently linked to the emergence of infectious zoonotic diseases [29–31]. Therefore, considering the role of the environment is critical for the assessment of MHC gene variability.

Bats (*Chiroptera*) are the second largest species-rich mammalian order, accounting for 20 % of all mammal species in the world. Compared to other mammals, their uncommon biological features (e.g., ability to fly, long lifespan, complex social structures) and their long-term evolutionary history with pathogens make them excellent reservoirs and spreaders of viruses (e.g., rabies virus, henipaviruses, coronaviruses), which have a significant impact on both human and animal health [32]. However, bats rarely exhibit clinical or pathological signs of diseases and appear to coexist with their pathogens in a disease-free state, characteristic of reservoir hosts [33]. At the order level, the monophyly of *Chiroptera* was evidenced *via* phylogenetic relationships inferred from intron sequences of *DRB* [34]. The monophyletic origin of *DRB* genes was also evidenced at the family level *via* phylogenetic relationships inferred from *DRB* sequences from *Saccopteryx bilineata*, *Myotis* spp*.*, *Noctilio* spp*.* and *Carollia perspicillata* [35]. Other studies, investigating the diversity of MHC *DRB*, showed significant differences in the polymorphism of MHC genes between species. This polymorphism was essentially influenced by (1) a pathogen-driven selection [36], (2) a social structure driven by MHC-mediated post-copulatory mechanisms [37], (3) diversifying selection and recombination events [35, 38] and (4) geographical constraints resulting in spatial variation of pathogen-mediated selection and enhanced susceptibility to environmental changes [39, 40].

The Amazon, a major biodiversity hotspot in South America, possesses a large diversity of bat species and pathogens in a wide variety of climates and vegetation formations [41, 42]. French Guiana, a tropical Amazonian region near the equatorial zone, harbors a great diversity of bats, with 103 species registered [43]. In this region, the impact of deforestation on the composition and dynamics of bat communities was assessed [44, 45] as well as, the role of habitats on rabies virus circulation and maintenance [46].

In this study, we analyzed the genetic variability of the expressed MHC class II *DRB* exon 2 in three bat species: two Phyllostomidae, *Carollia perspicillata* and *Desmodus rotundus*, and one Molossidae, *Molossus molossus*. These three widely distributed species have ecological plasticity and tolerance to disturbance. In French Guiana, these species are sympatric and use the same habitats (e.g., forests, edge habitats, and urban areas).

The key hypothesis of this study was that composition and distribution of MHC *DRB* alleles were specific to the environments (forests *vs.* disturbed areas), rather than randomly distributed in space. Consequently, we should observe local immunogenetic adaptation to the contrasting pathogen pressures or equally adapted alleles. To assess which are the best factors that predict the MHC diversity, pathogen-mediated selection, recombination, gene conversion, demographic history and population structure were investigated.

There is a higher diversity of microorganisms in forest environments, compared to disturbed environnments, due to greater host species richness and better transmission-promoting parameters [47, 48]. For this reason, we expect higher levels of MHC diversity in forest environments facing lower disturbance pressures, where higher parasite and pathogen diversities imply a higher selection pressure. Furthermore, assuming that bats using the same roosting area and/or the same foraging areas would be subjected to similar pathogen pressures, we should observe similar trends in intra- and inter-specific MHC diversity. In contrast, once the demographic neutral genetic histories—which may also influence MHC diversity—are controlled, we should observe differences in selective histories between bats inhabiting different environments.

To identify different signatures of selection in the *DRB* exon 2, which would imply area-specific recognition capabilities, conformation of the *HLA*-*DRB*1 was used as a reference to detect putative sites directly involved in antigen binding (ABS) and consequently subjected to selection. Additionally, to reduce the misidentification bias of ABS, the selection signature within the PBR was investigated without *a priori* identifying species-specific ABS for further comparisons.

MHC genes exhibit high levels of allele similarity within species as well as between related species and the occurrence of identical MHC alleles in related species is frequent. Convergence and trans-species polymorphism are thought to be responsible for this trans-species evolution. To highlight which of these two mechanisms acts predominantly on the evolutionary history of the *DRB* gene in the three species investigated, phylogenetic relationships were inferred from the *DRB* sequences obtained here and with other available chiropteran sequences. Finally, MHC spatial diversity was compared to that of neutral markers (mtDNA D-loop) to highlight the impact of demographic processes and population structure on the diversity pattern in the three bat species investigated.

## Methods

### Ethics statement

Animals were captured, handled, sampled and, whenever necessary, euthanized following ASM guidelines [49] under the supervision of researchers who had been granted the French “Expérimentation animale niveau 1” diploma. Bats are not protected by law in French Guiana; however, the project was submitted to the Conseil Scientifique Régional pour le Patrimoine Naturel de la Guyane and approved. Captures that occurred within protected areas (nature reserves) received approval by the Conseil Scientifique Régional du Patrimoine Naturel on 26 January 2010 and ad-hoc authorizations (no. 2011-35 dated 05/30/2011, no. 35 and 59 obtained on 03/21/2013 and 04/17/2013, respectively, and delivered by the Prefecture of French Guiana).

### Study areas, sample collection, DNA and RNA extraction

**Fig. 1:**
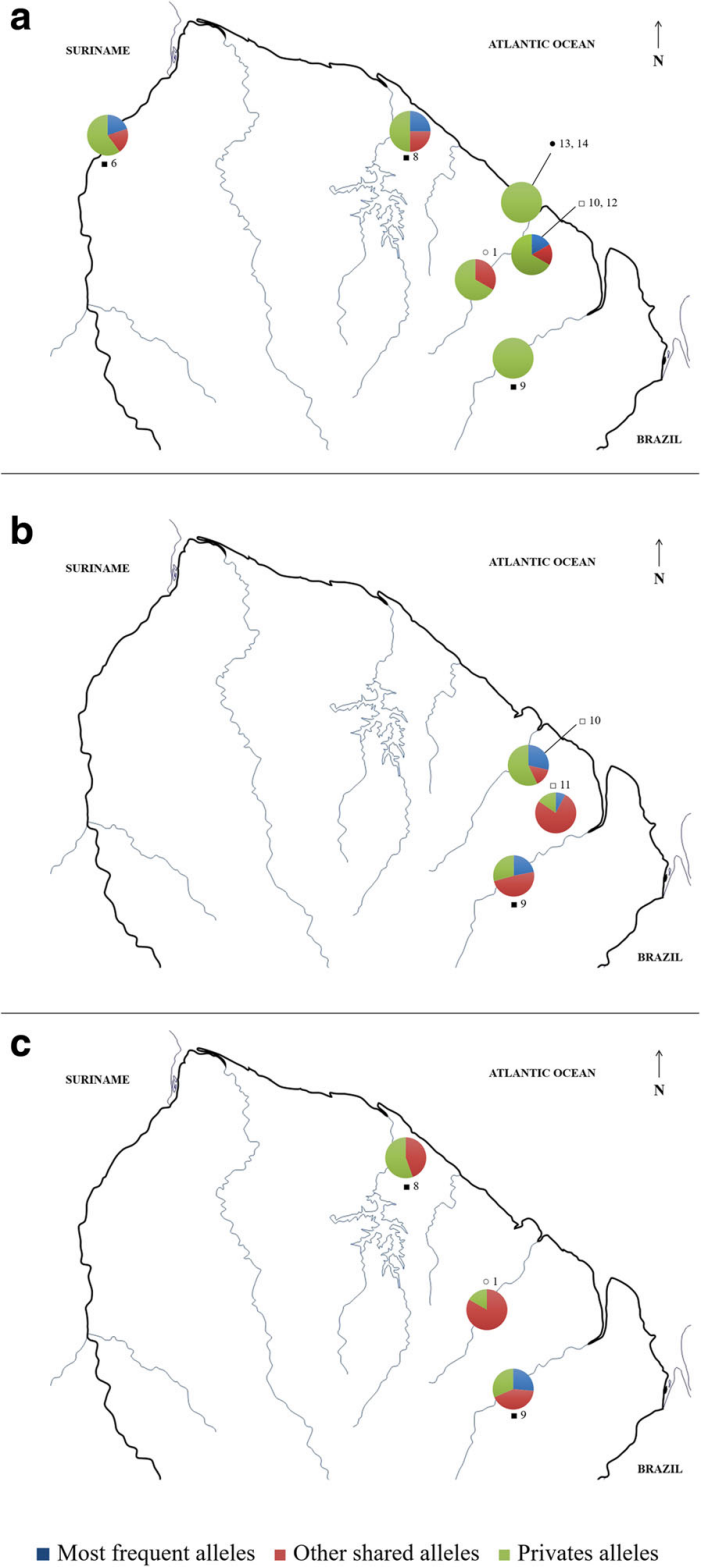
Map of sampling sites of all individuals used in this study across four environments. For clarity, nearby collecting sites within 15 km were grouped. Sites are numbered and labeled according to the type of environment to which they correspond: edge habitats (*dark square*), anthropized areas (light circle), pristine primary lowland forests (*light square*), urban and periurban areas (*dark circle*). Pie charts indicate the proportion of the most frequent alleles (alleles with the highest overall frequencies: *Cape*-DRB*16 for *C. perspicillata* (**a**); *Dero*-DRB*02 and *19* for *D. rotundus* (**b**); Momo-DRB*08 for *M. molossus* (**c**)), other shared alleles and private alleles (those detected only in one individual) in each collecting sites/environments, for each species: *C. perspicillata* (**a**), *D. rotundus* (**b**) and *M. molossus* (**c**)

The three bat species (*C. perspicillata*, *D. rotundus* and *M. molossus*) were sampled at 14 sites. Three sites were located in pristine lowland forests (PF sites) with low disturbance pressure, four sites were located in edge habitats (EH sites), five in anthropized areas (AA sites), and two in urban and periurban areas (UR sites) (Fig. 1, Additional file 1: Table S1 and Additional file 2: Figure S1).

Bats were trapped with mist nets erected near breeding sites, roosts, at forest edges, around livestock, or through putative foraging courses. Bat species were identified in the field using external morphology and, prior to release, a brachial vein puncture and wing biopsies were performed. A few individuals captured were euthanized at the laboratory to collect organs. A total of 45 *C. perspicillata*, 59 *D. rotundus* and 42 *M. molossus* were sampled.

Nucleic acids were extracted from blood, liver or spleen depending on sampling. Extractions were performed using the NucliSENS easyMAG bio-robot (bioMérieux®).

### Amplification and genotyping of the MHC class II DRB gene

All amplifications of MHC *DRB* genes for the three species were performed using cDNA obtained from liver and spleen RNAs. All cDNAs were synthesized using SuperScript® III Reverse Transcriptase (Invitrogen) and random hexamers (Roche) following the manufacturer’s recommendations. Expressed MHC *DRB* genes of the three bat species were amplified by a minimum of two independent polymerase chain reactions (PCRs) using a standard procedure with the AmpliTaq Gold DNA Polymerase PCR kit (Thermo Fisher Scientific). To optimize the detection of different alleles, a minimum of two distinct PCRs per individual were performed. Species-specific primers were designed for *C. perspicillata* and *D. rotundus* to avoid occurrence of non-amplifying alleles (Table 1). To amplify the *M. molossus DRB* gene, primers designed by Richman *et al.* [39] were modified. Primers located in conserved parts of exon 1 (JSex1.5, [37]; EX1Fd, modified from [39]), exon 2 (JS2Cape, [37]), exon 3 (EX3Rd, modified from [39]) and exon 4 (L729, [50]) were used to amplify functional MHC class II *DRB* alleles from the cDNA of three bats from each species, *C. perspicillata*, *D. rotundus* and *M. molossus*. The sequences obtained were used to design specific primers complementary to conserved parts of exon 1 (BatCMHIIF (F) and BatCMHIIF.1 (F1), Table 1) and exon 4 (BatCMHIIR (R) and BatCMHIIR.1 (R1), Table 1). The primer combinations F-R and F1-R1 were used as the first PCR for *D. rotundus* and *C. perspicillata* (Additional file 3: Figure S2). For the second PCR, all of the newly designed primers were used together with JSex1.5, EX1Rd, EX3Rd and L729 in different combinations to screen the cDNA of a total of 24 *C. perspicillata* and 39 *D. rotundus* (Additional file 3: Figure S2). Two different primer combinations with JS2Cape, EX3Rd, R and R1 were used to amplify the *DRB* alleles from the cDNA of 40 *M. molossus* (Additional file 3: Figure S2). The PCR products were cloned using the pCR™4-TOPO® TA Cloning® Kit (Invitrogen). Ten clones per PCR amplification were sent for sequencing to Beckman Coulter Genomics (Takeley, UK) using T7 and T3 primers.

**Table 1:** PCR primers used to amplify MHC class II DR *beta* loci in the three species investigated: *C. perspicillata*, *D. rotundus* and *M. molossus*

Primers	Code^a^	Position^a^	5′ to 3′ Sequence	Designed for	Source
BatCMHIIF	F	Exon 1	GATHCTGTTGGYACTGAGC	*D. rotundus*	This study
BatCMHIIF.1	F1	Exon 1	TGGTGATMCTGTTGGCACTGAGC	Bats	This study
EX1Fd^b^	X1	Exon 1	CTGWTGGYACTGAGCCCTCYCCTGGCT	*Myotis* sp.	Richman et al. [39]
JSex1.5-*DRB*	JS1	Exon 1	KGGGCCAGRGRCACACCA	Bats	Schad et al. [35]
JS2Cape-*DRB*	JS2	Exon 2	AGTGTCAKTWCTCCAACSGGAC	*C. perspicillata*	Schad et al. [35]
EX3Rd^b^	X3	Exon 3	CAGSAGGTTGTGGTGCTGCAG	*Myotis* sp.	Richman et al. [39]
BatCMHIIR	R	Exon 4	TTCAGACTGYGCCCTCCAYT	*D. rotundus*	This study
BatCMHIIR.1	R1	Exon 4	CAGACTGYGCCCTCCAYTCCA	Bats	This study
DRB-L729	L729	Exon 4	ACTCAMCATCTTGCTCTG	Mammals	Bowen et al. [50]

^a^see Additional file: Figure S2, ^b^Primer modified from Richman *et al.* [39] to amplify the exon 2 of MHC class II *DRB* gene

### Amplification and sequencing of the mtDNA D-loop

All amplifications of mitochondrial DNA (mtDNA) control region (D-loop) for the three species were performed using genomic DNA. The primers F(mt) and P(mt) [51] were used to amplify the hypervariable domain HVI of mtDNA D-loop. PCRs were performed using a standard procedure with BIOTAQ™ DNA Polymerase PCR kit (Bioline). Sequencing was carried out by Beckman Coulter Genomics (Takeley, UK) using F(mt) and P(mt) primers.

### Sampling analysis

To assess the environmental impact on the MHC class II *DRB* diversity, the data were analyzed according to the location of the capture sites and the type of environment at these sites. For *D. rotundus* and *M. molossus*, each capture site corresponded to one environment type. *D. rotundus* sampling sites corresponded to two different roots (Cave F (CF) and Cave M (CM)), considered as PF sites, and one foraging area (Saut Athanase (SA)), an EH site (Fig. 1, Additional file 1: Table S1). *M. molossus* individuals were captured in one foraging area (Cacao (CC)), an AA site, and two different roosts located at the EH site with different disturbance levels: Paracou (PA) with a high level of disturbance and the SA site with a low level of disturbance (Fig. 1, Additional file 1: Table S1). *C. perspicillata* individuals were captured in the four types of environments PF, EH, AA and UR (Fig. 1, Additional file 1: Table S1). To explore the potential bias induced by the spatial structure of collecting sites in *C. perspicillata* samples, spatial structure was compared with environment structure.

### Data analysis

Sequences were edited using Geneious R6 [52]. DNA sequences were aligned using the Mafft alignment tool [53] included in the software. Sequences were confirmed as MHC class II *DRB* and mtDNA D-loop by homology analysis using the NCBI BLAST search [54]. A *DRB* clone sequence was regarded as valid if the following criteria were met: (1) incidence in at least two independent PCRs either from the same individual or different ones, or amplification by two different primer pairs, and (2) identified by at least three identical clones [55]. Haplotypes were reconstructed for each bat species using the DnaSP 5 [56]. As proposed by [57], the nomenclature of MHC alleles for non-human species was used: to each allele a prefix (the first two letters of the genus and the species names) was given, with the serial number attached as follows: *Cape*-*DRB* for *C. perspicillata*, *Dero*-*DRB* for *D. rotundus* and *Momo-DRB* for *M. molossus. Cape*-*DRB* alleles identified in this study were named after the 15 alleles previously described by [35].

### Sequence diversity

Allele frequencies (*F*), proportion of segregating sites (*S*), nucleotide diversity (π), observed (*H*_*O*_) and expected (*H*_*E*_) heterozygosities, and gene diversity (*H*_*D*_) were estimated using Arlequin 3.5 [58]. Allelic richness (*R*), with a correction for sample size using a rarefaction method and inbreeding coefficients (F_IS_) were calculated using Fstat 2.9. [59]. The mean number of differences between nucleotide and amino acid alleles were counted using MEGA 6.06 [60]. Analyses were performed per species for the entire dataset, and for each environment subgroup separately.

### Gene conversion and recombination events

Evidence of genetic recombination or gene conversion events between *DRB* sequences was assessed using GENECONV 1.81a [61]. This program detects lengthy patterns shared between sequences despite the existence of a pronounced polymorphism. *P-*values were determined from both global and pairwise permutation tests, with 10,000 replicates. No mismatches between fragments were accepted, and global *p*-values were corrected for multiple comparisons (gscale = 0). Recombination breakpoints were identified using the GARD and SBP algorithms [62] implemented in the *HyPhy* package [63], available in the standard Datamonkey analysis library (http://www.datamonkey.org/, Delport et al. [64]).

### Codon-based analysis of positive selection

Putative antigen-binding sites (ABS) were identified by comparison with the human ABS of the *HLA-DRB1* [65]. The relative rates of d_N_ and d_S_ substitutions were calculated for putative ABS, non-ABS and all codons following the method of Nei and Gojobori with the Jukes and Cantor correction for multiple hits [66, 67]. A Z-test was performed to assess the deviation of the d_N_/d_S_ (ω) ratio from 1.0. These tests were performed using MEGA 6.06.

Codons potentially subjected to positive selection were further identified with no *a priori* assumption of ABS. For this purpose, two maximum-likelihood (ML) frameworks were carried out as proposed by [68]. First, two alternative models comprised in the CodeML Program included in the PAML package version 4.7 were executed [69]. The first one (M7) allows codons to evolve either neutrally (ω = 1) or under purifying selection (ω < 1), while the second one (M8) considers a class of sites under positive selection (ω > 1). These models were compared with a likelihood ratio test (LTR; 2Δl = 2-l_1_-l_0_) with two degrees of freedom (α = 0.05) [70]. As previously described, each analysis was run twice, with starting ω values of 0.5 and 1.5, to ensure convergence [68]. The F3 × 4 model of codon frequencies was assumed for all analyses. The amino acids positively selected were identified using the Bayes empirical Bayes approach (BEB), as recommended by Yang [71], with the cutoff posterior probability set at 90 %. Secondly, five ML methods proposed in the *HyPhy* package were used. For all analyses, the best fitting nucleotide substitution model was assessed with the automatic model selection tool available on the server. A single likelihood ancestor counting (SLAC) model was used to detect evidence of the non-neutral evolution of the *DRB* gene [72]. Based on ancestral reconstruction, this model counts d_N_ and d_S_ changes at each codon position in a phylogeny. Fixed (FEL) and random effects likelihood (REL) models were performed to estimate the ω ratio on a site-by-site basis [72]. For the FEL model, ω ratios were estimated with an *a priori* distribution of rates across sites, while this distribution was determined by the present data for the REL model. An internal FEL (IFEL) model was carried out to highlight selection pressure at the population level (i.e., along internal branches). Finally, a mixed effects model of evolution (MEME) was performed to highlight both diversifying and importantly episodic selection at individual sites [73]. Codon positions were regarded as candidates for positive selection if the following two criteria were met: (1) *p*-values < 0.1 for SLAC, FEL, IFEL and MEME and (2) Bayes Factor > 50 for REL. Accordingly, only sites identified as being under positive selection by at least two approaches were considered [68]. Analyses were performed per species for the entire dataset and for each environment subgroup separately.

### Genetic differentiation and past population dynamics

For both neutral and functional markers, the genetic differentiation (*F*_ST_) among environment subgroups and collecting sites was calculated through an implementation of analysis of molecular variance (AMOVA; 10,000 permutations) using Arlequin 3.5.

Isolation-by-distance (IBD) was examined with the web service IDBWS v.3.23 (http://ibdws.sdsu.edu/~ibdws/; [74]) by assessing the statistical significance of the correlation in a Mantel test with 30,000 permutations. The quantity *F*_ST_/(1 – *F*_ST_) was used as a genetic distance according to Rousset [75]. Geographic distances between populations were determined from GPS coordinates recorded at each collecting site.

To add a measurement of relationships between alleles at the intraspecific level, haplotype networks were constructed for each species using the parsimony statistical algorithm TCS implemented in PopArt 1.7 [76].

A Bayesian skyline plot was used to investigate changes in effective population size (Ne) over time with Beast 1.8.1 [77], using mtDNA D-loop data only. Markov Chain Monte Carlo samples were based on 2 × 10^7^ generations, logging every 200 steps, with the first 2,000,000 generations discarded as the burn-in. The best-fitted model HKY + G was used with a relaxed molecular clock. The number of stepwise changes in Ne was set at five.

### MHC class II *DRB* phylogeny

Phylogenetic relationships and trans-species polymorphism between *DRB* alleles were inferred using a Bayesian inference approach implemented in MrBayes 3 [78]. The *HLA-DRB1* sequence (accession number: NG_029921) was used as outgroup. The GTR + G + I model was selected as the best-fitted model of nucleotide substitution using jModelTest 2 [79, 80] under corrected Akaike information criteria (AICc). The program was run 10 × 10^7^ generations, with a sampling frequency of 500 and a 25 % burn-in. Validation of the inference was assessed based on the standard deviation of split frequencies, less than the expected threshold value of 0.01.

## Results

### *DRB* sequence characterization

One hundred and three bats (24 *C. perspicillata*, 39 *D. rotundus* and 40 *M. molossus*) were tested for the amplification of MHC *DRB* using cDNA. PCR product sizes ranged from 357 bp to 600 bp, for both *C. perspicillata* and *D. rotundus* and covered all of exon 2 (267 bp in length) (Additional file 3: Figure S2). For *M. molossus*, we successfully amplified 244 bp, excluding primers, of exon 2. We successfully obtained 674 MHC *DRB*-like fragments from 97 bats (148 MHC *DRB*-like fragments for 22 *C. perspicillata*, 311 for 36 *D. rotundus* and 215 for 39 *M. molossus*, Additional file 1: Table S2). Based on BLAST searches, sequences showed homologies to only *DRB* loci.

Considering validation criteria, 480 sequences were validated, corresponding to 82 bats (Additional file 1: Table S2). One hundred and ten sequences were validated for 19 *C. perspicillata*, corresponding to 23 *Cape*-*DRB* alleles (previously reported *Cape*-DRB*05, GenBank accession number: JQ388834, and *Cape*-DRB*16-*37*; GenBank accession numbers: KU896612–KU896633; Additional file 1: Table S3). Two hundred and thirty-three sequences were validated for 35 *D. rotundus*, corresponding to 30 *Dero*-*DRB* alleles (*Dero*-DRB*01–*30*; GenBank accession numbers: KU896562–KU896591; Additional file 1: Table S4). One hundred and forty-seven sequences were validated for 28 *M. molossus*, corresponding to 20 *Momo*-*DRB* alleles (*Momo*-DRB*01–*20*; GenBank accession numbers: KU896592–KU896611; Additional file 1: Table S5). All amino acid sequences were unique.

A maximum of three transcribed alleles per individual was detected in one *C. perspicillata* and two *D. rotundus* individuals (Additional file 1: Tables S3 and S4). No more than two transcribed alleles were identified in *M. molossus* samples (Additional file 1: Table S5).

All sequences showed BLAST homology with the *HLA-DRB1* locus (accession number: NG_029921), with a maximum of 85 % nucleotide identity and 75 % amino acid identity. The percentage of nucleotide and amino acid identity with other bat species (*S. bilineata*, *M. davidii*, *C. perspicillata* and *N. albiventris*) was above 87 % and 75 % for all transcripts, respectively.

### Detection of indels

**Fig. 2:**
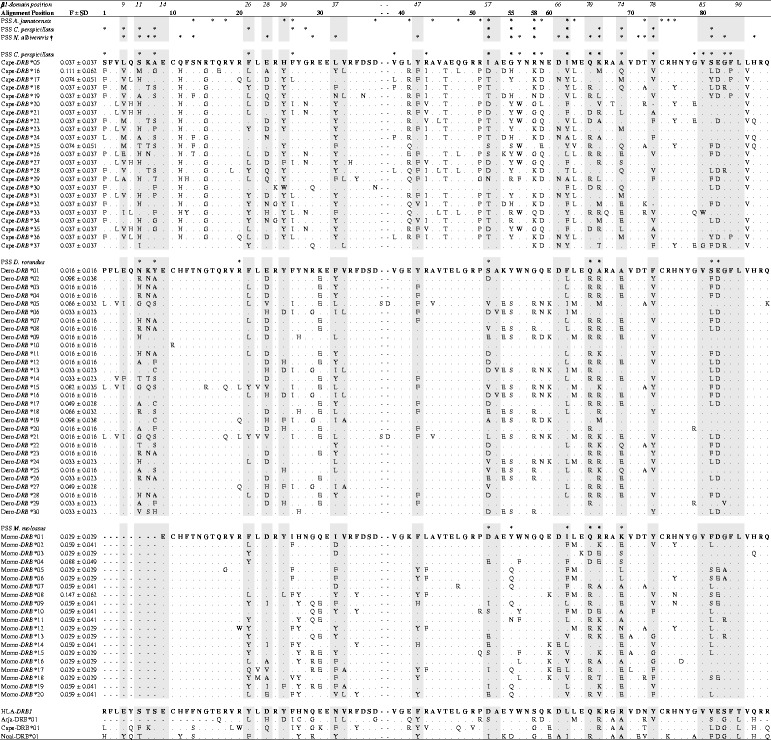
Amino acid sequence variation and overall frequency of MHC class II DR *beta* exon 2 alleles of *C. perspicillata*, *D. rotundus* and *M. molossus. DRB* sequences of *Homo sapiens* (accession number: NG_29921), *C. perspicillata* (Cape-DRB*01, accession number: JQ388830), *N. albiventris* (Noal-DRB*01, accession number: HM347941) and *A. jamaicensis* (Arja-DRB*01, accession number: KJ010995) are given for comparison. Antigen-binding sites (ABS) of the *HLA-DRB1* molecule are shadowed [65]. Dots mark identity with the top sequence. *Numbers in italics* indicate the amino acid positions within the *beta* 1 domain of the *HLA*-*DRB1* molecule. *Asterisks* indicate the positively selected sites (PSS) identified in each species, according to acceptance criteria described in the Methods section [68]. † indicates PSS identified by [35], and ‡ indicates PSS identified by [38]

Two alleles (*Cape*-DRB*20 and *32*) presented a 3-bp deletion at position 217–219 of the nucleotide alignment (Fig. 2). These single-codon indels did not induce a frame-shifting mutation or a stop codon. Bats carrying these alleles were heterozygotes and found in EH and UR environments. Two other alleles (*Dero*-DRB*05 and *Dero*-DRB*21) presented a 6-bp insertion at position 115–120 of the alignment. These two-codon indels – one serine (S) and one asparagine (D) – did not alter the reading frame. *Dero*-DRB*05 was carried by four bats, all captured in SA, with one homozygote. *Dero*-DRB*21 was carried by one heterozygote bat captured in CF. No indel event was detected for *M. molossus*.

### mtDNA D-loop sequence characterization

We amplified the first hypervariable segment (HVI) of the mitochondrial DNA (mtDNA) control region (D-loop) for the 146 bats tested. We obtained a 291-bp alignment for *M. molossus*, a 340-bp alignment for *C. perspicillata* and a 359-bp alignment for *D. rotundus*. Thirty-nine mtDNA D-loop haplotypes were identified for *C. perspicillata* (45 individuals, Genbank accession numbers: KU896735–KU896779), 29 for *M. molossus* (42 individuals, Genbank accession numbers: KU896693–KU896734) and only five for *D. rotundus* despite the high number of sequenced individuals (59 individuals, Genbank accession numbers: KU896634–KU896692) (Table 2).

**Table 2:** The MHC class II DR *beta* (exon 2) allelic diversity in 19 *C. perspicillata*, 35 *D. rotundus* and 28 *M. molossus*

								Mean # differences ± SD						
Species		Site	n	#	R	S	π ± SD	Nucleotide	Amino acid	H_O_	H_E_	H_D_	F_IS_	*p*-value
*C. perspicillata*	MHC	All	19	23	23.000	114	0.140 ± 0.070	37.387 ± 3.154	25.249 ± 2.477	0.368	0.963	0.986	0.624	0.0001
		UR	5	8	6.576	81	0.132 ± 0.074	34.536 ± 3.341	24.036 ± 2.772	0.600	0.86	1.000	0.400	0.0191
		EH	11	11	6.211	97	0.142 ± 0.074	38.145 ± 3.368	26.200 ± 2.697	0.182	0.884	0.974	0.667	0.0001
		PF	3	6	6.000	79	0.143 ± 0.084	38.133 ± 3.671	25.667 ± 2.754	0.667	0.778	1.000	0.745	0.1969
	D-LOOP	All	45	39	38.873	69	0.048 ± 0.024	16.439 ± 1.995	-	-	-	0.993	-	-
		AA	16	16	8.458	58	0.051 ± 0.027	17.375 ± 2.260	-	-	-	1.000	-	-
		UR	5	4	4.000	31	0.046 ± 0.029	15.600 ± 2.961	-	-	-	0.9	-	-
		EH	16	16	8.458	52	0.050 ± 0.026	16.967 ± 2.506	-	-	-	1.000	-	-
		PF	8	7	6.242	43	0.048 ± 0.027	16.393 ± 2.723	-	-	-	0.964	-	-
*D. rotundus*	MHC	All	35	30	22.371	71	0.105 ± 0.051	24.451 ± 2.733	16.722 ± 2.374	0.649	0.963	0.9679	0.330	0.0001
		SA	23	22	6.855	75	0.995 ± 0.049	24.697 ± 2.750	16.775 ± 2.367	0.640	0.936	0.957	0.334	0.0001
		CM	8	10	6.467	55	0.087 ± 0.046	24.556 ± 2.927	16.156 ± 2.522	0.625	0.883	0.9615	0.352	0.0005
		CF	4	7	7.000	69	0.112 ± 0.066	29.952 ± 3.370	21.190 ± 2.817	0.750	0.844	1.000	0.250	0.1696
	D-LOOP	All	59	5	4.913	8	0.008 ± 0.005	2.936 ± 1.561	-	-	-	0.67	-	-
		SA	22	5	4.334	8	0.007 ± 0.004	2.520 ± 1.410	-	-	-	0.576	-	-
		CM	27	3	2.853	6	0.008 ± 0.005	2.735 ± 1.497	-	-	-	0.581	-	-
		CF	10	2	2.000	1	0.001 ± 0.001	0.200 ± 0.269	-	-	-	0.200	-	-
*M. molossus*	MHC	All	28	20	18.351	70	0.102 ± 0.051	23.595 ± 2.729	15.847 ± 2.410	0.214	0.945	0.945	0.777	0.0001
		SA	15	12	6.606	57	0.092 ± 0.047	23.091 ± 2.751	15.333 ± 2.429	0.267	0.883	0.924	0.875	0.0001
		PA	8	9	7.375	57	0.089 ± 0.049	21.611 ± 2.622	13.889 ± 2.277	0.125	0.942	1.000	0.705	0.0001
		CC	5	5	5.000	51	0.102 ± 0.060	26.600 ± 3.253	17.500 ± 2.728	0.200	0.867	0.933	0.789	0.0035
	D-LOOP	All	42	25	25.000	55	0.041 ± 0.021	11.856 ± 1.845	-	-	-	0.977	-	-
		SA	22	16	9.045	35	0.031 ± 0.016	8.879 ± 0.638	-	-	-	0.97	-	-
		PA	14	12	8.648	32	0.033 ± 0.018	9.604 ± 1.070	-	-	-	0.978	-	-
		CC	6	5	5.000	20	0.030 ± 0.019	8.733 ± 1.087	-	-	-	0.933	-	-

The sample size (*n*), number of alleles (#), allelic richness (R), number of segregating sites (S), nucleotide diversity (π ± standard deviation), number of nucleotide and amino acid differences (± standard error) per sequence from averaging overall sequence pairs, observed (H_O_) and expected (H_E_) heterozygosities, gene diversity (H_D_), inbreeding coefficients (F_IS_) and their *p*-values are given for each species and defined subgroups. – computations not performed

### *DRB* allelic diversity

Overall, *C. perspicillata* presented the highest values of allelic richness (*R* = 23.000), number of segregating sites (*S* = 114), nucleotide diversity (π = 0.140 ± 0.070), gene diversity (*H*_*D*_ = 0.986) and mean nucleotide and amino acid differences (37.387 ± 3.154 and 25.249 ± 2.477, respectively), while *M. molossus* exhibited the lowest values (Table 2). The highest observed heterozygosity (*H*_*O*_) was recorded in *D. rotundus* and, as for the two other species, *H*_*O*_ was lower than expected heterozygosities (*H*_*E*_). Considering environment subgroups, the highest value of *R* was detected in UR for *C. perspicillata*, (R = 6.576), in CF for *D. rotundus* (R = 7.0), and in PA for *M. molossus* (R = 7.375). S ranged from 51 in CC for *M. molossus* to 97 in EH for *C. perspicillata*. The highest values of π were found in PF for *C. perspicillata*, CF for *D. rotundus* and CC for *M. molossus* (Table 2). F_IS_ values were high in all species as well as in all the subgroups per species, with values ranging from 0.330 (*p* <0.001) for *D. rotundus* to 0.777 (*p* <0.001) for *M. molossus*.

Recombination tests did not reveal fragments involved in gene conversion, recombination events or breakpoints among individuals from the same species or within a given environment or between species.

### mtDNA D-loop allelic diversity

*C. perspicillata* had the highest allelic richness values (*R* = 38.873), number of segregating sites (*S* = 69), gene diversity (*H*_*D*_ = 0.993) and mean nucleotide differences: 16.439 ± 7.460 (Table 2). Nucleotide diversity, for each species, was low (<0.05 in *C. perspicillata* and *M. molossus*, <0.01 in *D. rotundus*). Considering environment subgroups, π values ranged from 0.001 ± 0.001 in CF (*D. rotundus*) to 0.051 ± 0.027 in AA (*C. perspicillata*). The highest *R* values were detected in SA (*M. molossus* and *D. rotundus*), AA and EH (*C. perspicillata*). *S* ranged from one in CF (*D. rotundus*) to 58 in AA (*C. perspicillata*), and *H*_*D*_ ranged from 0.200 in CF (*D. rotundus*) to 1.0 in AA and EH (*C. perspicillata*).

### Distribution of *DRB* alleles across the different environments

#### *C. perspicillata*

Three alleles (*Cape*-DRB*16, *17*, and *25*) were shared either between bats trapped in the same environment (EH) or between bats trapped in distinct environments (EH *vs.* PF). These three alleles were the most common, with an overall frequency (F ± SD) of 0.111 ± 0.062 for *Cape*-DRB*16 and 0.074 ± 0.051 for both *Cape*-DRB*17 and *Cape*-DRB*25 (Fig. 2). *Cape*-DRB*16 was shared between one bat trapped in PF (*n* = 3) and two bats trapped in EH. *Cape*-DRB*17 was shared between two bats trapped in EH (*n* = 11). Nine individuals in this region were homozygotes (Additional file 1: Table S3). *Cape*-DRB*25 was shared between two bats trapped in PF and EH. Alleles found in UR were private alleles and three of the five trapped bats were heterozygotes.

#### *D. rotundus*

Up to 14 alleles were shared either between bats roosting in the same area or between bats foraging in the same area. Out of these alleles, one (*Dero*-DRB*19) was shared between six bats from the three sampling sites (CF, CM, and SA sites) (Additional file 1: Table S3). Five alleles (*Dero*-DRB*13, *17*, *18*, *27* and *30*) were shared between bats from SA and CM sites, and two alleles (*Dero*-DRB*02 and *15*) between the SA and CF sites. *Dero*-DRB*02 and *19* had the highest overall frequency of 0.085 ± 0.037 (Fig. 2). Four alleles (*Dero*-DRB*05, *06*, *14* and *29*) were exclusively shared between individuals trapped in SA. Two alleles (*Dero*-DRB*24 and *26*) were solely shared between individuals trapped in CM. We did not find shared alleles between bats trapped in CF (Additional file 1: Table S4). Sixteen of the 23 bats captured in SA were heterozygotes, five in CM (*n* = 8) and three in CF (*n* = 4).

#### *M. molossus*

Up to ten shared alleles were identified but none was shared between the three sampling sites (Additional file 1: Table S5). Three alleles (*Momo*-DRB*02, *04* and *19*) were shared between bats captured in the SA and PA sites. Two alleles (*Momo*-DRB*09 and *10*) were shared between individuals from the SA and CC sites. One allele (*Momo*-DRB*07) was shared between two bats captured in the CC and PA sites. Three alleles (*Momo*-DRB*08, *11* and *20*) were exclusively shared within SA. Momo-DRB*08 had the highest overall frequency: 0.147 ± 0.062 (Fig. 2). Four of the five bats trapped in CC were homozygotes, seven in PA (*n* = 8), and 11 in SA (*n* = 15).

### Detection of selection signature

An excess of d_N_ substitutions was detected for the three species (d_N_/d_S_ = [2.049–3.548], Z = [2.600–5.619], *p* = [<10^-5^-0.01] – Table 3). More specifically, in putative antigen binding sites (ABS), d_N_/d_S_ values were highly significant (*p* < 10^-3^) and ranged from 4.081 for *D. rotundus* to 4.939 for *C. perspicillata*. We found significant d_N_ substitutions also occurring in putative non-ABS for *C. perspicillata* (d_N_/d_S_ = 2.792, Z = 3.283, *p* = 0.0013) but none in both *D. rotundus* and *M. molossus* samples (*p* > 0.40).

**Table 3:** Non-synonymous (d_N_) and synonymous (d_S_) substitutions (± standard deviation) for *C. perspicillata*, *D. rotundus* and *M. molossus*

Species	Region	N	d_N_ ± SD	d_S_ ± SD	d_N_/d_S_	Z-test	*p-value*
*C. perspicillata*							
	ABS	25	0.405 ± 0.131	0.082 ± 0.064	4.939	5.119	<10^-5^
	Non-ABS	64	0.121 ± 0.047	0.043 ± 0.029	2.792	3.283	0.0013
	All	89	0.187 ± 0.053	0.0528 ± 0.026	3.548	5.619	<10^-5^
*D. rotundus*							
	ABS	25	0.314 ± 0.116	0.077 ± 0.055	4.081	3.811	0.0002
	Non-ABS	64	0.049 ± 0.035	0.045 ± 0.032	1.075	0.156	0.8764
	All	89	0.114 ± 0.470	0.054 ± 0.032	2.119	2.713	0.0077
*M. molossus*							
	ABS	21	0.323 ± 0.103	0.068 ± 0.058	4.753	4.080	0.0001
	Non-ABS	60	0.060 ± 0.021	0.056 ± 0.031	1.078	0.186	0.8528
	All	81	0.120 ± 0.028	0.058 ± 0.030	2.049	2.599	0.0105

Putative ABS and non-ABS were identified assuming functional homology to the human ABS of the *HLA-DR1* molecule Brown et al. [65]. N is the number of codons in each sequence region. Z-test values and *p-*values are shown

PAML analyses (performed by species and within subgroups per species) showed significant support for M7 and M8 (log likelihood estimates (LTR), 2Δl = [63.985–123.085], df = 2, *p* < 0.0001), indicating an effect of positive selection acting on the *DRB* gene. Overall, 22 codons appeared to be under positive selection for *C. perspicillata*, eight for *D. rotundus*, and six for *M. molossus* (BEB posterior probability *p* ≥ 90 %, SLAC, FEL, IFEL and MEME *p*-values < 0.1, and REL Bayes Factor > 50; Table 4). Three positively selected sites (PSS 52, 65 and 66; Fig. 2) were common to the three species investigated. These codons were the only common between *D. rotundus* and *M. molossus*. Six of the eight PSS identified in *D. rotundus* were also found in *C. perspicillata*, and all of the PSS identified in *M. molossus* were identified in *C. perspicillata*.

**Table 4:**
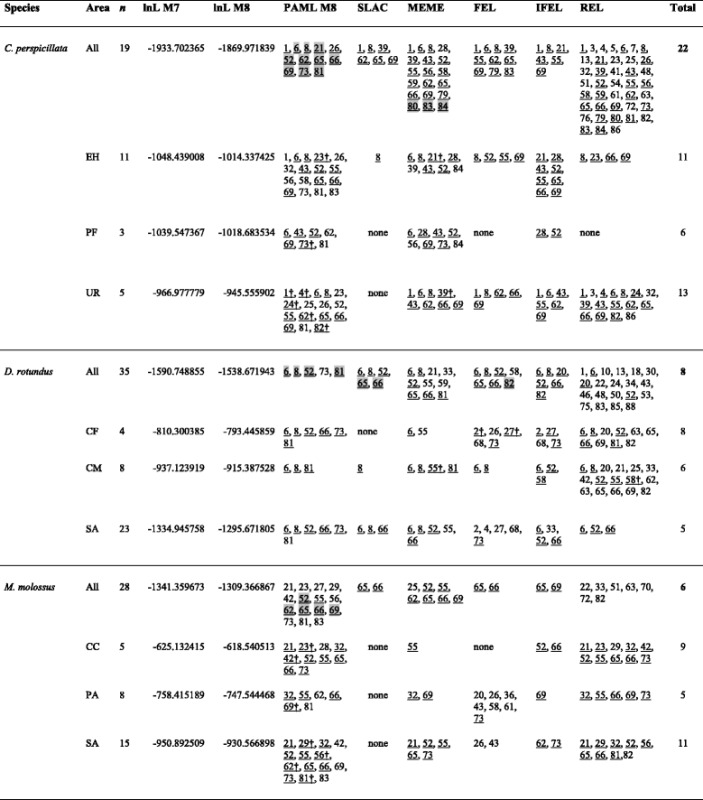
Identification of species-specific positively selected sites (PSS) by maximum likelihood (ML) analysis

Sample size (n) considered for each analysis is shown. Sites identified as positively selected by at least two ML analyses are underlined. Codons are given with a posterior probability ≥ 90 % in the Bayes Empirical Bayes (BEB) analysis for PAML M8; *p*-values < 0.1 for SLAC, FEL, IFEL and MEME; Bayes Factor > 50 for REL. † indicates PSS found only in a specific region for each species. Validated PSS also found with human antigen-binding sites (ABS) are shadowed

Regarding environment subgroups for all species, a maximum of 13 PSS was identified in UR for *C. perspicillata*, eight in CF for *D. rotundus* and 11 in SA for *M. molossus*. Three PSS were found in all environment subgroups for *D. rotundus*, four in *C. perspicillata* and four in *M. molossus*. A maximum of six unique PSS (sites detected as positive only within the defined subgroup) was observed for *C. perspicillata*, four for *M. molossus* and two unique PSS were observed in the two roosts (CF and CM) for *D. rotundus* (Table 4). None of the PSS detected in *C. perspicillata* was shared between UR and PF. The same result was obtained between SA and CM for *D. rotundus* and between PA and CC or SA for *M. molossus*. Thirteen of the 22 PSS identified were located within the human ABS region for *C. perspicillata*, seven for *D. rotundus* and five for *M. molossus* (Fig. 2). All the PSS identified in *M. molossus* were described as PSS for *N. albiventris*, 13 for *C. perspicillata* and six for *D. rotundus*. Six PSS in *C. perspicillata* were also found in *A. jamaicensis*, three for *M. molossus* and one for *D. rotundus*. The sites where PSS differed were located within one to six amino acid residues (3–18 bp) of the human ABS or other bat species PSS, depending on the bat species investigated.

### *DRB* population structure

**Fig. 3:**
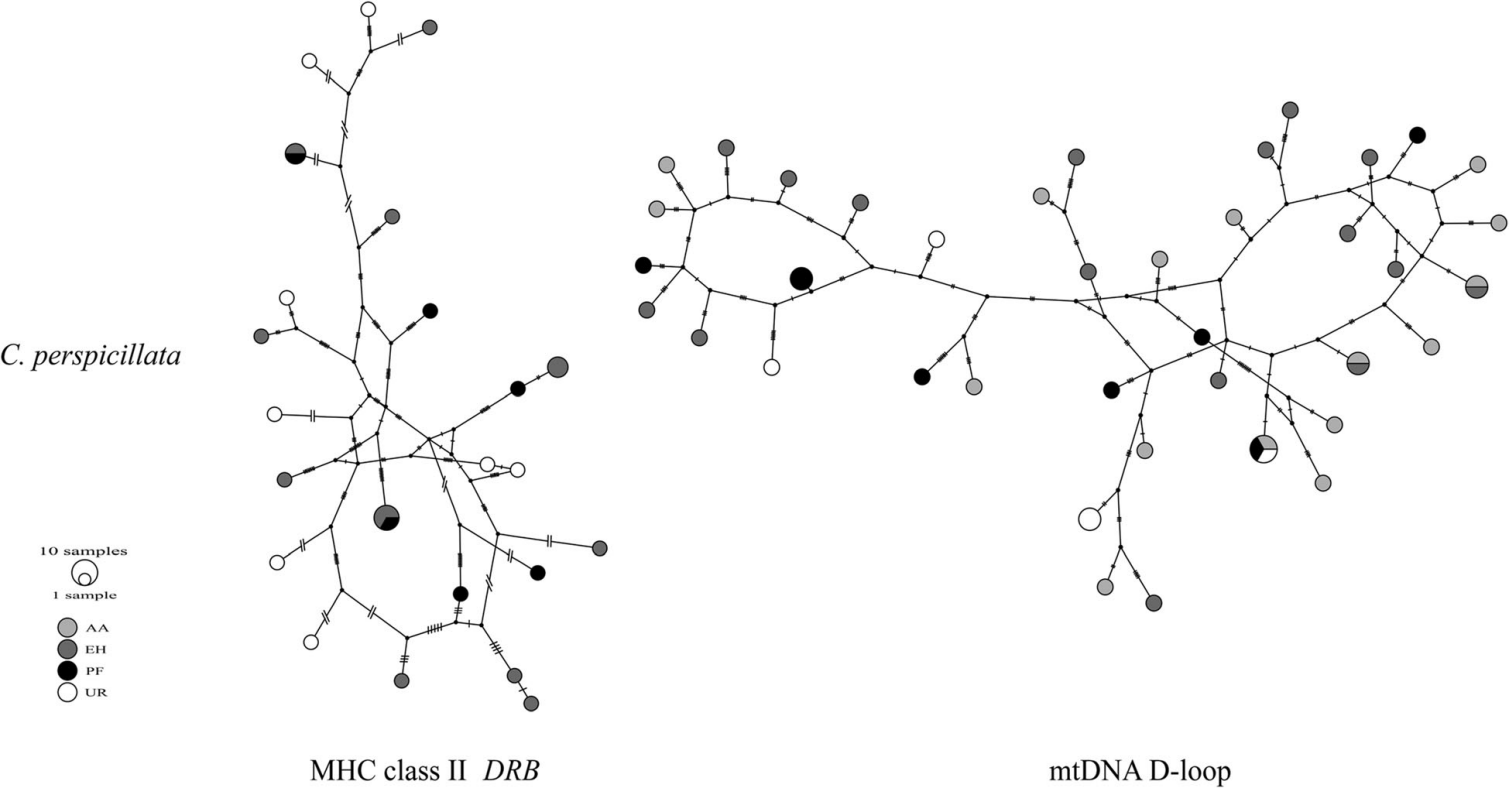
Haplotype networks of MHC class II DR *beta* exon 2 alleles and of the mitochondrial DNA (mtDNA) control region (D-loop) at the nucleotide level for *C. perspicillata*. Nodes are proportional to the number of bats carrying each haplotype, colored by the environments where the bats were trapped (see legend). Hatch marks represent mutations. Interruptions in *lines* indicate the presence of more than ten mutations

**Fig. 4:**
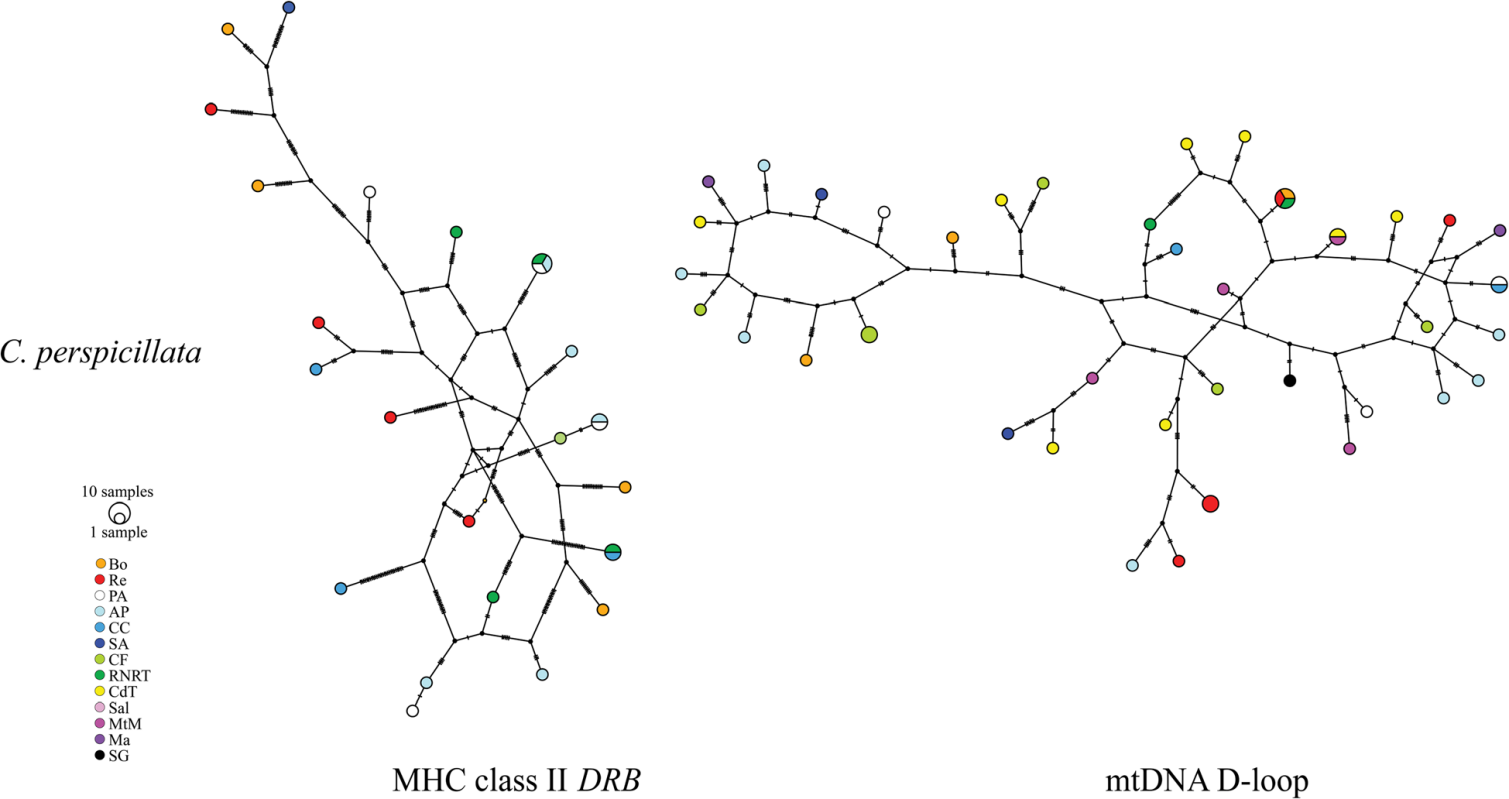
Haplotype networks of MHC class II DR *beta* exon 2 alleles and of the mitochondrial DNA (mtDNA) control region (D-loop) at the nucleotide level for *C. perspicillata* based on collecting sites. Nodes are proportional to the number of bats carrying each haplotype, *colored* by the capture site where the bats were trapped (see legend). *Hatch marks* represent mutations

**Fig. 5:**
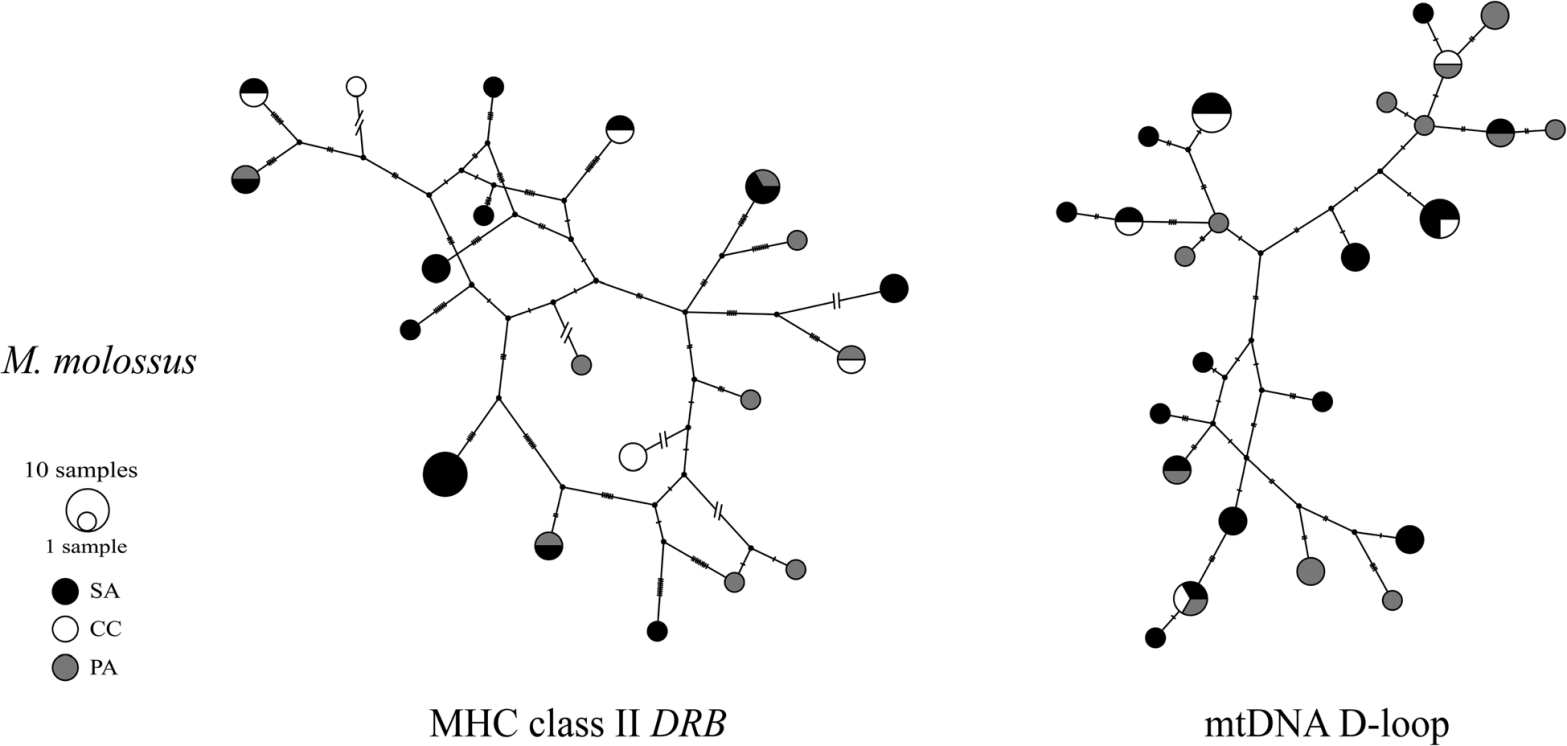
Haplotype networks of MHC class II DR *beta* exon 2 alleles and the mitochondrial DNA (mtDNA) control region (D-loop) at the nucleotide level for *D. rotundus*. Nodes are proportional to the number of bats carrying each haplotype, *colored* by the capture site where the bats were trapped (see legend). *Hatch marks* represent mutations. Interruptions in *lines* indicate the presence of more than ten mutations

**Fig. 6:**
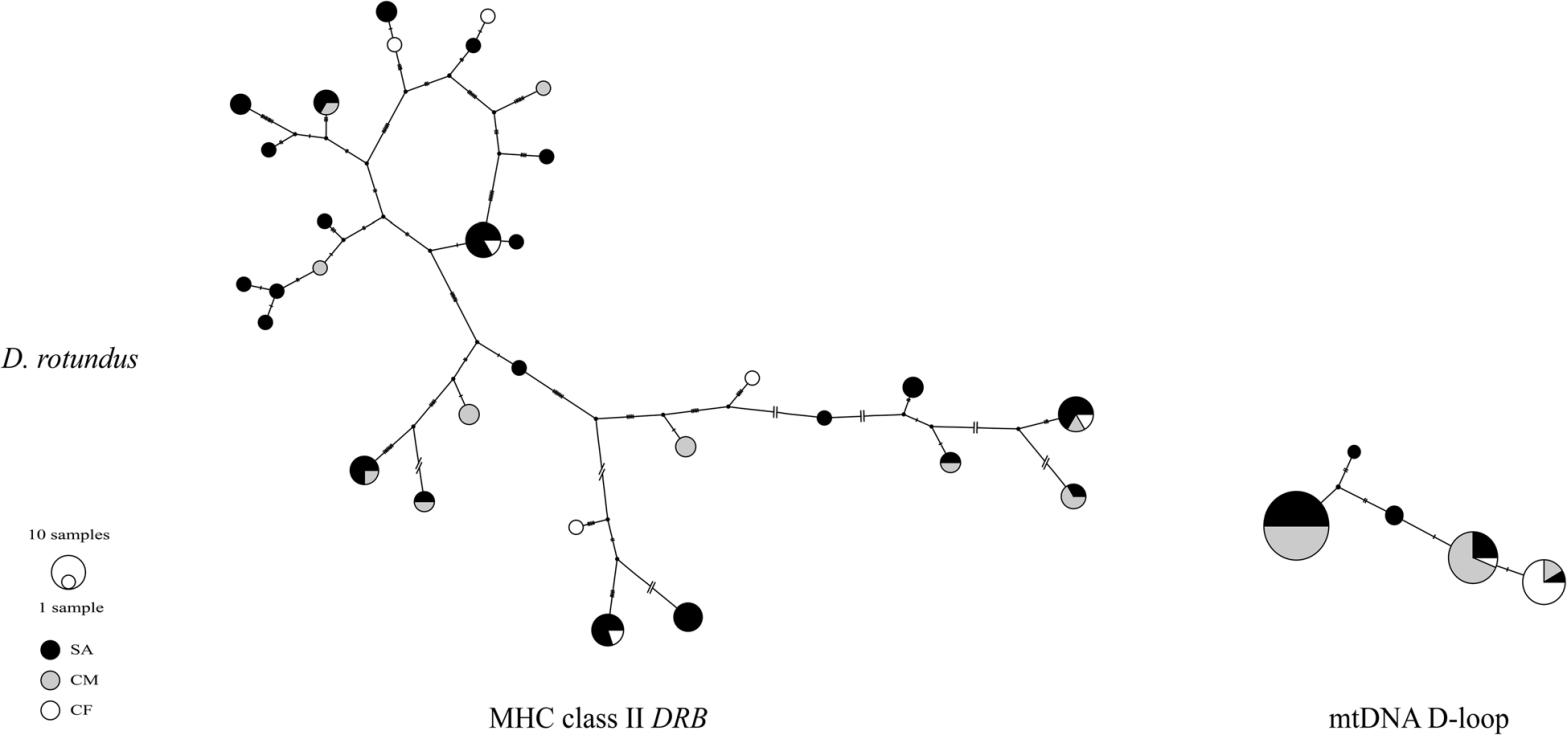
Haplotype networks of MHC class II DR *beta* exon 2 alleles and of the mitochondrial DNA (mtDNA) control region (D-loop) at the nucleotide level for *M. molossus.* Nodes are proportional to the number of bats carrying each haplotype, *colored* by the capture site where the bats were trapped (see legend). *Hatch marks* represent mutations. Interruptions in *lines* indicate the presence of more than ten mutations

We found no significant genetic differentiation between *DRB* alleles of the environment subgroups defined for each species (*C. perspicillata*: F_ST_ range: [(−0.068) to 0.042]; *D. rotundus*: F_ST_ range: [(−0.047) to 0.012]; *M. molossus*: F_ST_ range: [0.013–0.055], Additional file 1: Table S6(A) − 8). Taking into account the collecting sites, pairwise differentiation tests did not reveal significant genetic differentiation between *DRB* alleles (*C. perspicillata*: F_ST_ range: [(−0.048) to 1.000]; *D. rotundus*: F_ST_ range: [(−0.047) to 0.012]; *M. molossus*: F_ST_ range: [0.013–0.055], (Additional file 1: Table S6(B) − 8). Minimum spanning trees showed complex connections between the haplotypes, marked by numerous nucleotide mutations, without clustering of haplotypes either by site or by environment (Figs. 3, 4, 5 and 6). We did not find relevant isolation-by-distance in our samples (Additional file 1: Table S9).

### mtDNA D-loop population structure

We found significant genetic differentiation between some populations of *D. rotundus* (CF *vs.* CM F_ST_: 0.475, *p*-value <0.0001; CM *vs.* SA F_ST_: 0.617, *p*-value < 0.0001; but CM *vs.* SA F_ST_: 0.016, *p*-value not significant). No significant genetic differentiation was observed between populations for the two other species (*C. perspicillata* F_ST_ range: [(−0.029) to (−0.016)]; *M. molossus* F_ST_ range: [(−0.040) to 0.017], Additional file 1: Table S6(A)–8). Pairwise computations performed between collecting sites did not reveal significant genetic differentiation (*C. perspicillata* F_ST_ range: [0.072 to 1.000]; *M. molossus* F_ST_ range: [(−0.040) to 0.017], Additional file 1: Table S6(B)–8). The haplotype networks for *C. perspicillata* revealed two main groups of haplotypes that possessed all the haplotypes found in the different environments or collecting sites (Figs. 3 and 4). The same result was obtained for *M. molossus*, with a maximum of four mutations between haplotypes (Fig. 5). The lowest number of mutations was found for *D. rotundus* for which the five haplotypes were linked by one connection, marked by only one or two mutations (Fig. 6). No population expansion or collapse was observed for the three species. We did not find relevant isolation-by-distance in our samples (Additional file 1: Table S7).

### *DRB* phylogenetic relationships

**Fig. 7:**
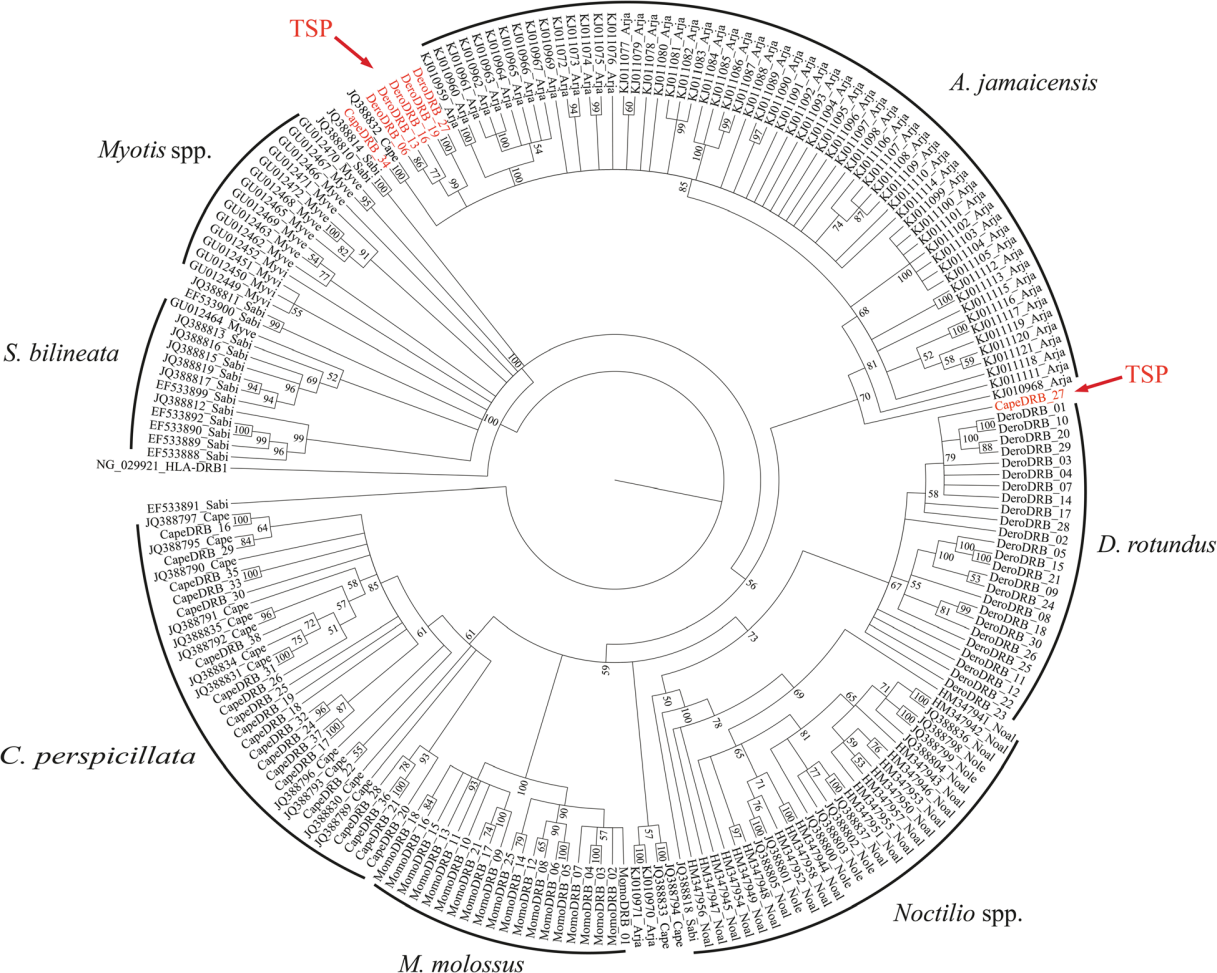
Phylogenetic relationships of the MHC class II DR *beta* sequences based on part of exon 2 (174 bp) with values of posterior probabilities for nodes. Species designation follows the GenBank accession numbers for previously described *DRB* sequences. Arja *A. jamaicensis*, Cape *C. perspicillata*, Dero *D. rotundus*, Momo *M. molossus*, Myve *M. velifer*, Myvi *M. vivesi*, Noal *N. albiventris*, Nole *N. leporinus*, Sabi *S. bilineata. HLA-DRB1* was used as the outgroup. *TSP* indicates trans-species polymorphism of the newly described *DRB* alleles with previously described *DRB* alleles

The analysis included the 73 *DRB* sequences identified in this study (174 bp; nucleotide position 48–219), as well as the 63 published chiropteran *DRB* sequences selected randomly from *A. jamaicensis* [38], 15 from *C. perspicillata* [35], 15 from *Myotis* spp. (*M. velifer* and *M. vivesi* [39]), 28 from *Noctilio* spp. (*N. albiventris* and *N. leporinus* [35, 37]) and 17 from *S. bilineata* [35, 36]*.* The phylogenetic tree showed six major clades (Fig. 7). We observed a clustering by species or genus supported by medium and high posterior probabilities – *C. perspicillata* (0.61), *D. rotundus* (0.67), *M. molossus* (1), *Noctilio* spp. (0.50) – with an intermingled clustering of *N. albiventris* and *N. leporinus DRB* alleles. Within the *C. perspicillata* clade, *DRB* sequences clustered with the previously published sequences, with a high posterior probability (0.85). One *DRB* sequence of *C. perspicillata* was related to a *D. rotundus* clade with a posterior probability of 0.79. One *DRB* sequence from *S. bilineata* clustered with the clade *Noctilio* spp*.*, with a low posterior probability value. The 63 *DRB* sequences of the *A. jamaicensis* species clustered with five *DRB* sequences of *D. rotundus* and two *DRB* sequences of *C. perspicillata*, with a posterior probability of 0.70. Within this phyllostomid clade, we observed a species-dependent clustering with high posterior probabilities. Two *DRB* sequences of *C. perspicillata* clustered with a low posterior probability with two sequences from *A. jamaicensis*. We did not observe a characteristic clustering for *S. bilineata* and *Myotis* spp. However, one *DRB* sequence from *M. velifer* clustered together with six *DRB* sequences from *S. bilineata* with a low posterior probability.

## Discussion

In this study, we explored the genetic variability of the expressed MHC *DRB* genes of three sympatric Neotropical bat species, looking for selection signatures within the PBR and investigating the role of the environment and the population structure on MHC diversity.

### Diversity patterns in the MHC class II *DRB* gene

In the three species, identified alleles (23 in *C. perspicillata*, 30 in *D. rotundus* and 20 in *M. molossus*) were unique in amino acid sequences, suggesting a non-redundant nucleotide polymorphism. The number of alleles detected per species and per environment subgroup within each species indicated a relatively high level of MHC *DRB* variation. These results are in agreement with previously reported allelic copy number variation between or within bat species [1, 35–40].

This high *DRB* polymorphism was observed in the three species whatever the degree of habitat disturbance (heavily disturbed, slightly impacted or pristine). In addition, no loss of polymorphism induced by low host and/or pathogen diversity, expected to occur in disturbed environments [47, 48] was observed. Furthermore, mtDNA D-loop sequences, considered as neutral, revealed high levels of genetic diversity for *C. perspicillata* and *M. molossus.* High levels of neutral diversity have been associated with either multiple geographic origin and/or population admixture [81, 82], suggesting that *C. perspicillata* and *M. molossus* populations originated from one of these two phenomena or from both. The high level of *DRB* polymorphism can be related to population history for these two species with the effects of additional selection factors such as pathogen-mediated selection.

A high number of *DRB* homozygotes was also found in *M. molossus* in all habitats as well as in *C. perspicillata* but in edge habitats only*.* For these two species, the presence of a specific allele (*allele-specific overdominance*) ensuring an adequate immunity can be hypothesized [1]. Considering *D. rotundus*, a high number of heterozygotes for *DRB* was observed in all habitats. This result suggests a heterozygote advantage related to the forest (pristine and edge habitats) environment known to encompass higher species and pathogen richness [83, 84].

The most frequent MHC *DRB* alleles encountered in each bat species (*Dero*-DRB*02, *Dero*-DRB*15, Dero-DRB*19, Cape-DRB*16 and Momo-DRB*08) were all identified in forest environments. These alleles are likely ancient and play a significant role in the immune response, conferring a selective advantage to the bats in sympatry in these habitats. Indeed, given that forest environments are characterized by a high level of pathogen diversity, these alleles most likely possess enhanced recognition capabilities and might be able to recognize a broader range of pathogens. They thus allow a more efficient immune response in line with the ecological diversity. Moreover, the geographical structure highlighted in *D. rotundus*, using mtDNA D-loop results, did not affect the allelic distribution. Indeed, most of the alleles identified in *D. rotundus* were shared between sites, and one allele (*Dero*-DRB*19) was present at all sites. In contrast, no geographical structure was found in *C. perspicillata* even if the alleles identified in urban and periurban areas were rare and not shared with other groups. These results suggest that environment rather than population structure drives the allelic distribution, corroborating a local adaptation hypothesis. Although edge and forest habitats may differ in pathogenic pressures (see above), the results observed for *C. perspicillata* may be related to the high tolerance of this species to habitat disturbance and to its dominance in bat communities in edge habitats [45], likely allowing continuous exposure to the communities of pathogens of both pristine and disturbed forests, and pathogen transfer between these habitats.

### Duplication detected in Phyllostomidae

Gene conversion and recombination events were not detected for any species. The identification of three expressed *DRB* alleles in individuals from *C. perspicillata* and *D. rotundus* supported the existence of at least two functional *DRB* loci. However, underestimation of the number of alleles and expressed loci can be suspected, considering the limited number of recombinant clones per primer combinations and the use of cDNA only allowing the detection of the most frequently expressed alleles. The occurrence of multiple MHC *DRB* loci varying in the number of loci between individuals and species was reported in Neotropical bat species such as *C. perspicillata* (three *DRB* loci), *S. bilineata* (ten *DRB* loci) [35], *A. jamaicensis* (three *DRB* loci) [38], as well as in other animals [1, 7, 85, 86]. Together with environmental and biological patterns, gene conversion and recombination processes are known to drive the extensive MHC polymorphism [18, 87].

Variable gene duplication between closely related taxa and among individuals from the same species is characteristic of MHC genes and plays a critical role in the adaptive evolution of the host [88]. Gene duplication can occur over different time scales and therefore predates or follows speciation events. Furthermore, the absence of recombination between alleles of different species was reported. This result was regarded as evidence of recent duplication events between loci that occurred after speciation [35]. Our findings support this hypothesis since some recent radiation events occurred in Neotropical bat species during the Pleistocene era (0.01–1.8 Mya) [89–91].

Despite the indels detected in some *C. perspicillata* and *D. rotundus* sequences, the reading frames remained unaltered, suggesting that these sequences encode functional proteins (Fig. 2). Nevertheless, the heterozygote-dominant character of individuals with these alleles suggests a turnover of these loci generated by evolutionary processes such as the silencing of duplicate genes by mutation or deletion [88]. Under the birth-and-death model, genes created by duplication can be maintained for long periods, deleted or transformed as pseudogenes and, therefore, contribute to host fitness and adaptability [92]. The phenomenon is especially significant among heterozygote *D. rotundus*, for which one of the two alleles is among the most widespread in the population studied (Dero-DRB*02, *05* and *15*), and supports a shift towards an allele with better recognition capabilities.

### Evidence of historical positive selection

Higher rates of non-synonymous (d_N_) *vs.* synonymous (d_S_) nucleotide substitutions were observed, especially in sites supposedly involved in antigen-binding. This excess of d_N_ over d_S_ in ABS is consistent with balancing selection acting on *DRB* loci, as a sign of historical positive selection in polymorphic MHC genes [93]. Furthermore, an unexpected excess of d_N_ in putative non-ABS for *C. perspicillata* suggests strong positive selection acting on these sites and highlights species-specific divergence on sites involved in antigen recognition.

A high congruence was observed between species-specific positively selected sites (PSS) in the three species and human ABS demonstrating the functional homology of these sites. In contrast, PSS not identified as human ABS might play a role in MHC *DRB* recognition capability or in the molecule stability for the species studied. Here, nine of the PSS were detected in *C. perspicillata*, while only one was detected in both *D. rotundus* and *M. molossus*. Therefore, the observed excess of d_N_ in *C. perspicillata* was attributed to PSS identified as non-ABS in humans. This result demonstrates the critical role of positive selection in shaping MHC diversity and corroborates that positive selection is driven by a species-specific immune response. Focusing on *C. perspicillata*, comparisons of PSS identified here with those previously described revealed high congruence, even among PSS not identified as human ABS [35]. A similar result was observed between *C. perspicillata* and *A. jamaicensis*, two Phyllostomidae with the same diet [38]. Taken together, these findings reveal family and species-specific selection pressures acting on MHC genes. Moreover, the high similarity of PSS between *A. jamaicensis* and *C. perspicillata* highlights that bat species sharing similar feeding strategies might be subjected – independently of their habitats – to similar pathogenic pressures, mainly from their microbiota [94]. Comparisons between each environment subgroup, for each present case, revealed the existence of private PSS depending on the area, suggesting a habitat-specific selection pressure process. Indeed, while six private PSS were counted in urban and periurban areas for *C. perspicillata*, only two were detected in edge habitats and one in pristine forest.

### Phylogeny, trans-species polymorphism and demographic process

Two mechanisms are thought to be responsible for the trans-specific similarity of MHC genes: convergent evolution [93] and trans-species polymorphism [95, 96]. Evidence of trans-species polymorphism in bats was reported in both *Myotis* spp. and *Noctilio* spp. [35] and at the family level between the two phyllostomids *A. jamaicensis* and *C. perspicillata* [38]. Phylogenetic analyses in our study did not reveal any characteristic clustering of MHC alleles depending on the habitat, but rather clades at the family, genus and species levels (Fig. 3). These results suggest either similar pathogenic pressures or that these alleles possess a larger antigenic recognition capability that constitutes a major asset to the immune response of these species. Intermingled clusterings observed in this survey (between *DRB* sequences from *A. jamaicensis*, *C. perspicillata* and *D. rotundus)* are in agreement with trans-species polymorphism reported in bats [35, 38]. The observed trans-species polymorphism for *Myotis* spp*.* [35] was not detected in the present study. The results highlighted clustering between *S. bilineata* and *M. velifer*. However, this clustering was no longer observed by analyzing full-length exon 2. Observation of trans-species polymorphism within bat families (e.g., Phyllostomidae) strengthens the scenario of independent modes of evolution of MHC *DRB* alleles, allowing balancing selection to retain substantial allelic lineages before speciation events [95, 96]. Extended periods of host–pathogen coevolution is thought to contribute to trans-species polymorphism given that host-sharing pathogens and exposure to similar pathogen pressures are believed to induce similar changes in different host species [97].

Although pathogen-driven selection is known to be crucial in MHC diversity [98], demographic processes such as genetic drift, bottlenecks, expansions, fragmentation and geographic isolation have been demonstrated to also participate in the shaping of this extensive polymorphism [15, 17, 99, 100]. Evolutionary analyses of MHC *DRB* alleles for the three species did not reveal any area-dependent clustering effect. Furthermore, analyses of partial sequences of the mtDNA D-loop revealed no structuration for *C. perspicillata* and *M. molossus*, whereas it was observed for *D. rotundus*. Demographic analysis performed for the three species did not detect any substantial bottleneck or expansion. While the patterns observed for the first two species corroborate the hypothesis of a similarity between the intrinsic genetic diversity of the species and that of MHC, the lack of similarity between these patterns of diversity for *D. rotundus* contradicts this statement. These results suggest that the signature of an area-limited pathogen-driven selection, supported by the presence of private PSS rather than by demographic processes such as distance isolation, is observed in this case.

## Conclusions

This study was the first to investigate MHC *DRB* polymorphism in three sympatric bat species, *M. molossus*, *C. perspicillata* and *D. rotundus*, from the Amazonian region. Our results revealed a high genetic variability in the MHC *DRB* gene. Natural selection, as well as local adaptation driven by different parasite and pathogen exposures across environments, contribute to the maintenance of an extensive MHC polymorphism. The richness of pathogens in the different habitats should be investigated to strengthen our assumptions on potential local adaptation. Further studies using finer neutral markers such as microsatellites will be necessary to detect possible confounding effects, such as hidden structuring and demographic history, and thus to better understand the drivers of MHC allelic diversity.

## Additional files

Additional file 1: Table S1. Supplementary methods, including the characteristics of all capture sites (Table S1)﻿, the list of samples successfully amplified for the MHC class II *DRB *loci (Table S2), the distribution of *DRB *alleles per species (Table S3-5), the pairwise differentiation computations per species (Table S6-8), as well as the correlations of genetics and geographic distance (Table S9). (XLS 179 kb)
Additional file 2: Figure S1. Map of French Guiana showing the capture sites of *C. perspicillata*, *D. rotundus* and *M. molossus*. For clarity, nearby sites within 15 km were grouped. Sites are numbered and labeled according to the type of environment to which they correspond: edge habitats (dark square), anthropized areas (light circle), pristine primary lowland forests (light square), urban and periurban areas (dark circle). Pie chart indicates the proportion of bat species sampled, with *C. perspicillata* in light grey, *D. rotundus* in black and *M. molossus* in dark grey. Small charts indicate a total number of individuals caught ≤ 10, medium-sized charts (16-22 individuals) , large charts (34-61 individuals) . Characteristics of the different sites are given in Additional file 1: Table S1. (PDF 432 kb)
Additional file 3: Figure S2. Positions of PCR primers used to amplify the indicated fragments of the MHC class II loci in *C. perspicillata*, *D. rotundus* and *M. molossus*, based on cDNA. The structure of the cDNA of the MHC class II *DRB* gene is based on [50]. According to each species, boxes indicate the amplified region using each couple of primers. The shaded region represents the region of interest, namely exon 2. Sequences and references of all primers used are given in Table 1. (PDF 26 kb)

## Abbreviations

ABS: Antigen-binding sites
AICc: Corrected Akaike information criteria
BEB: Bayes empirical Bayes
DRB: Exon 2 of the MHC class II DR beta
FEL: Fixed effects likelihood
IFEL: Internal fixed effects likelihood
MEME: Mixed effects model of evolution
MHC: Major histocompatibility complex
ML: Maximum-likelihood
mtDNA D-Loop: Mitochondrial DNA control region
PBR: Peptide-binding region
PCR: Polymerase chain reaction
PSS: Species-specific positively selected sites
REL: Random effects likelihood
SLAC: Single likelihood ancestor counting
