# Supplementary figures and images for: Spatial pattern of genetic diversity and selection in the MHC class II DRB of three Neotropical bat species
Source: BMC Evol Biol. 2016 Oct 26;16:229. doi: 10.1186/s12862-016-0802-1 (PMC5080761; doi:10.1186/s12862-016-0802-1)

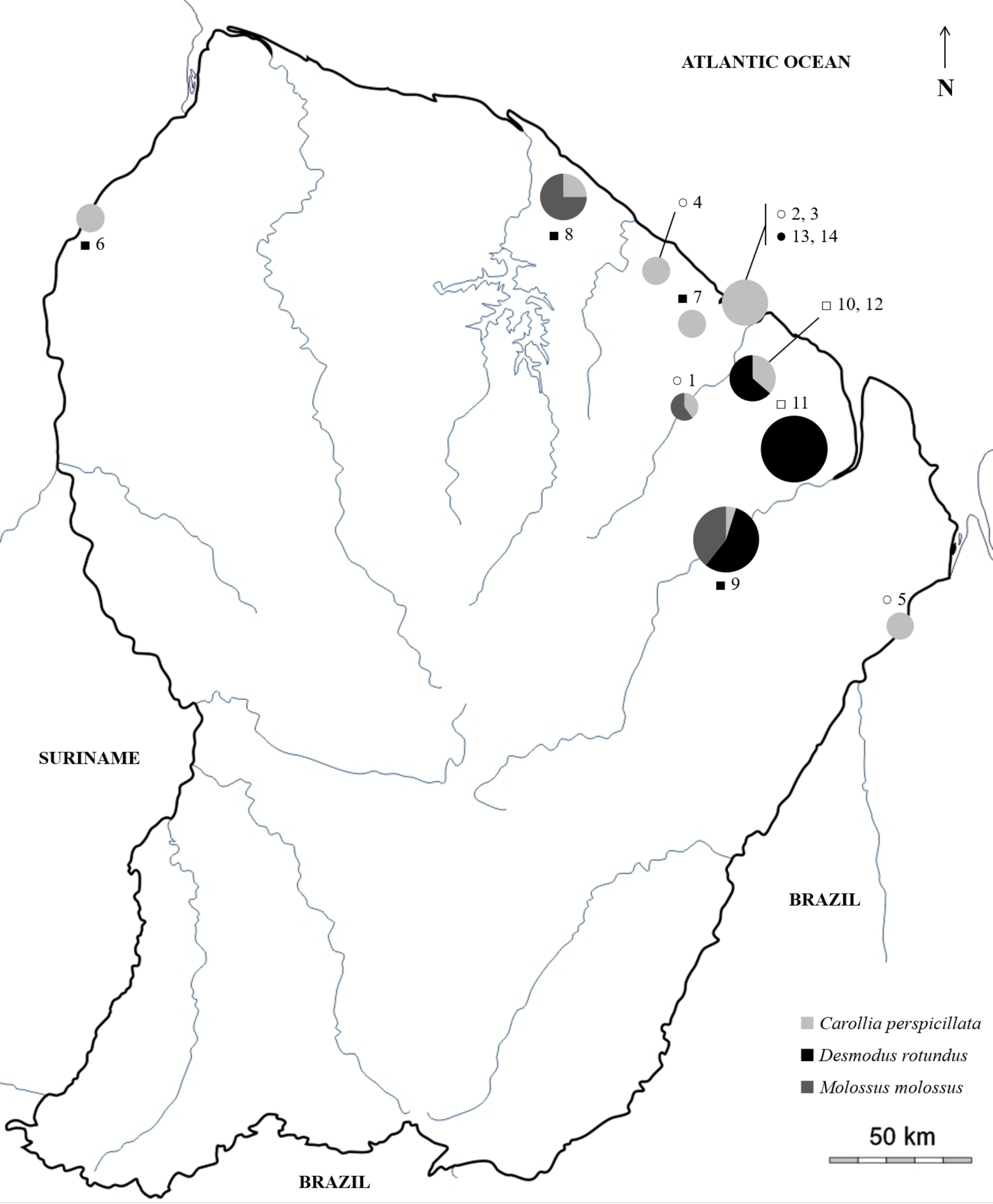

Supplement: Additional file 2: Figure S1. — Map of French Guiana showing the capture sites of C. perspicillata, D. rotundus and M. molossus. For clarity, nearby sites within 15 km were grouped. Sites are numbered and labeled according to the type of environment to which they correspond: edge habitats (dark square), anthropized areas (light circle), pristine primary lowland forests (light square), urban and periurban areas (dark circle). Pie chart indicates the proportion of bat species sampled, with C. perspicillata in light grey, D. rotundus in black and M. molossus in dark grey. Small charts indicate a total number of individuals caught ≤ 10, medium-sized charts (16-22 individuals) , large charts (34-61 individuals) . Characteristics of the different sites are given in Additional file 1: Table S1. (PDF 432 kb) [file 12862_2016_802_MOESM2_ESM.pdf]

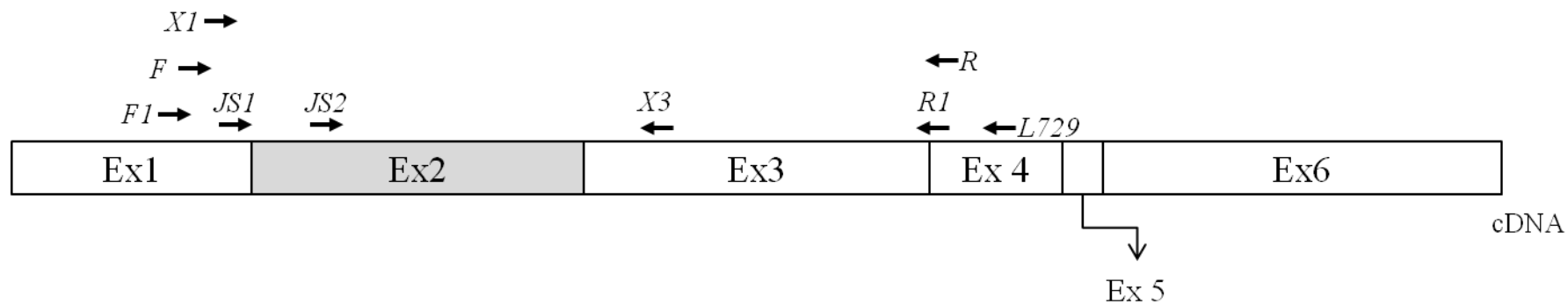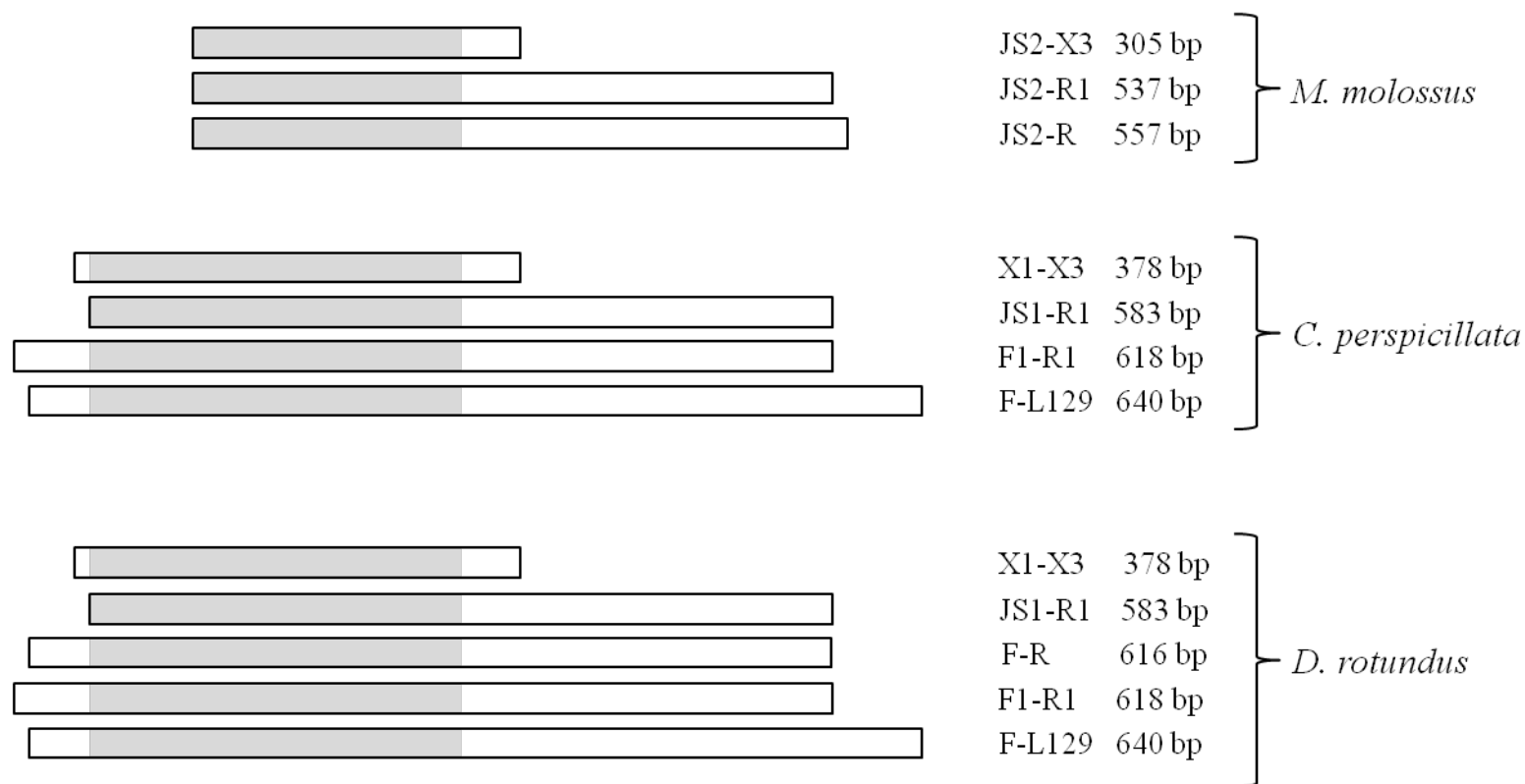

Supplement: Additional file 3: Figure S2. — Positions of PCR primers used to amplify the indicated fragments of the MHC class II loci in C. perspicillata, D. rotundus and M. molossus, based on cDNA. The structure of the cDNA of the MHC class II DRB gene is based on [50]. According to each species, boxes indicate the amplified region using each couple of primers. The shaded region represents the region of interest, namely exon 2. Sequences and references of all primers used are given in Table 1. (PDF 26 kb) [file 12862_2016_802_MOESM3_ESM.pdf]
